# An unusual presentation of macular telangiectasia type 2 with a large pigment deposit

**DOI:** 10.3205/oc000176

**Published:** 2021-01-28

**Authors:** Sugandha Goel, Purna Nangia, A. Joash Rijey, Kumar Saurabh, Rupak Roy

**Affiliations:** 1 Department of Vitreo Retina, Aditya Birla Sankara Nethralaya, Kolkata, West Bengal, India

**Keywords:** macular telangiectasia type 2, blue light reflectance, MacTel

## Abstract

Macular telangiectasia type 2 (MacTel) is a bilateral retinal disease that seems to be limited to the juxtafoveal region of the macula. We herein report an unusual presentation of MacTel with a large pigment deposit at the macula. Fundus of the right eye showed a large pigment deposit at the macula and right-angled venule. The left eye fundus showed a grayish discoloration due to subretinal fibrosis, dark pigment clumps and right-angled venule in the macula. Lesions were highlighted on multicolor imaging and blue reflectance imaging. Spectral domain optical coherence tomography (SD-OCT) of both eyes showed hyperreflectivity on the inner aspect of the retina corresponding to the area of pigment clumping.

## Introduction

Macular telangiectasia type 2 (MacTel) is a bilateral retinal disease that seems to be limited to the juxtafoveal region of the macula [1]. It is usually diagnosed in the fifth or sixth decade of life. Clinical features range from the patient being asymptomatic to having minimal visual disturbances. Impaired reading ability is the most common initial visual disturbance, or the patient may present with scotoma or metamorphopsia [2], [3]. We herein report an unusual presentation of MacTel with a large pigment deposit at the macula.

## Case description

**Figure 1:**
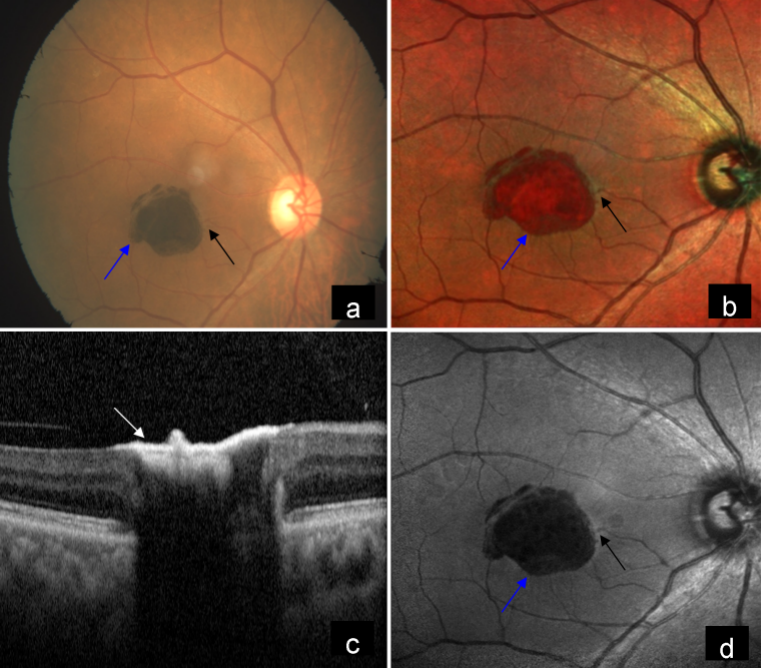
a) Color fundus photograph (CFP) of the right eye showed a large pigment deposit (blue arrow) and right-angled venule (black arrow) in the macula. b) Multicolor image (MCI) of the right eye showed pigmented lesion as orange-red in color with well-demarcated borders (blue arrow) and highlighted the right-angled venule clearly (black arrow). c) Spectral domain optical coherence tomography (SD-OCT) showed inner layer hyperreflectivity (white arrow). d) Blue reflectance (BR) showed decreased reflectance in the corresponding lesion (blue arrow) and highlighted right-angled venule (black arrow).

**Figure 2:**
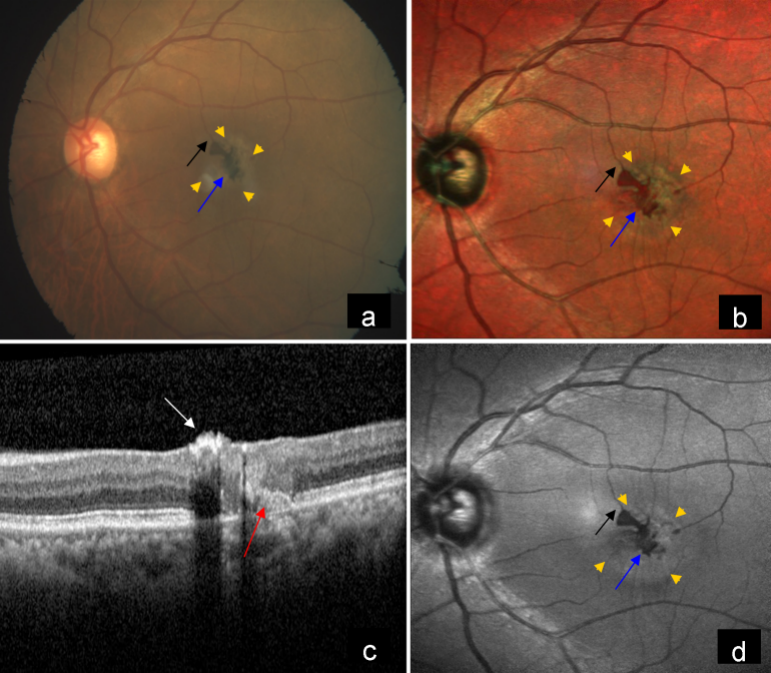
a) CFP of the left eye showed grayish discoloration at the macular area (yellow arrow heads) due to subretinal fibrosis, dark pigment clumps (blue arrow) and right-angled venule (black arrow). b) MCI of the left eye showed orange-red pigments (blue arrow) surrounded by grayish subretinal fibrosis (yellow arrow heads) and right-angled venule (black arrow). c) SD-OCT showed inner layer hyperreflectivity (white arrow), RPE proliferation and disruption of the ellipsoid zone and the interdigitation zone (red arrow). d) BR highlighted dark pigments (blue arrow), right-angled venule (black arrow) and picked up more areas of graying (yellow arrow heads).

A 65-year-old female patient came to our hospital with the complaint of diminution of vision in both eyes. Best corrected visual acuity was counting fingers at 2 meters in the right eye, and 20/30 in the left eye. The patient gave a history of diabetes and hypertension for 10 years. Fundus examination of the right eye showed a large pigmented lesion and right-angled venule in the macula (Figure 1a (Fig. 1)). Multicolor imaging (MCI) showed the pigmented lesion as orange-red in color with well-demarcated borders as well as non-visibility of retinal blood vessels under the lesion, and highlighted the right-angled venule clearly (Figure 1b (Fig. 1)). Spectral domain optical coherence tomography (SD-OCT) at the level of the lesion showed inner layer hyperreflectivity, non-visibility of the outer retina, choroid and altered foveal contour (Figure 1c (Fig. 1)). Blue reflectance (BR) showed decreased reflectance in the corresponding lesion and highlighted the right-angled venule (Figure 1d (Fig. 1)). Left eye fundus showed grayish discoloration due to subretinal fibrosis, dark pigment clumps and right-angled venule in the macula (Figure 2a (Fig. 2)). MCI of the left eye showed orange-red pigments surrounded by grayish subretinal fibrosis and right-angled venule (Figure 2b (Fig. 2)). SD-OCT showed inner layer hyperreflectivity, altered foveal contour, retinal pigment epithelium (RPE) proliferation, as well as disruption of the ellipsoid zone and the interdigitation zone (Figure 2c (Fig. 2)). BR highlighted dark pigments and right-angled venule and picked up more areas of graying (Figure 2d (Fig. 2)).

## Discussion

Reduced retinal transparency (“retinal graying”) in the parafoveolar area may be the first ophthalmoscopically visible change in patients with MacTel, which is seen as an area of increased reflectance on confocal BR imaging. Paracentral vertically oriented slightly dilated right-angled venules are seen draining the telangiectatic area. These right-angled venules typically develop temporally. It has been suggested that a retinal Muller glial dysfunction or cell death may contribute to the formation of these venules [4]. Due to RPE migration into the retina along the course of the right-angled venules, retinal pigmented epithelial hyperplasia or clumps may be seen around the parafoveolar right-angled venules, and these pigments may extend into the inner retina to form irregular plaques enveloping these venules [5]. This pigmentary “sheath” may also provide support for the abnormal blood vessels and increase the propensity of the tissue for fibrosis and tissue contraction [4]. Pigment clumping is initiated in the areas of outer retinal thinning and disruption, which suggests that atrophic changes in the photoreceptor layer may create a permissive environment for RPE transformation. Pigment clumps increase in size over time, and in some cases, separate pigment clumps can coalesce and form a large contiguous pigment clump [6]. Similarly, intraretinal migration of RPE can be noted in retinitis pigmentosa and intermediate dry age-related macular degeneration. Bone spicule pigments in retinitis pigmentosa are formed in areas devoid of photoreceptors. It has been suggested that direct contact between inner retinal vessels and RPE appears to be a major trigger for migration of RPE cells [7], [8]. In dry age-related macular degeneration, drusen may play physical and catalytic roles in facilitating intraretinal RPE migration [9]. The laser channel of MCI that images deeper lesions is infrared, and the pseudocolor assigned to this channel is red. Thus, pigment is visualized as orange-red in color on MCI.

Optical coherence tomography angiography (OCTA) is a novel technique that visualizes vascular tissue using flow characteristics. Volume-rendered OCTA allows clear visualization of retinal vasculature at all depths. Subretinal neovascularization can occur in MacTel. RPE abnormalities may provide a conduit for abnormal vessels in the subretinal space to proliferate into the sub-RPE compartment [10]. In OCT-A, right-angled vessels can be detected in early stages that can be tracked from superficial to outer retinal layers. They can form anastomoses in the outer retina with disease progression [11].

Extensive pigmentation can often be seen in patients treated with focal laser or photodynamic therapy. In our case, there was no previous history of laser treatment. Interestingly we found an unusual large dark pigment deposit at the macula which has not yet been described in the literature. However, these pigmented lesions should be differentiated from tumors of the RPE that include solitary congenital hypertrophy of the RPE (CHRPE), congenital hamartoma of the RPE, combined hamartoma of retina and RPE (CHRRPE) and adenoma of RPE. The differentiation between these conditions is primarily done clinically. CHRPE is a peripheral or mid-peripheral fundus lesion that is well-demarcated, flat or slightly elevated, and can either be black homogeneous with typical depigmented lacunae or completely depigmented [12]. Most solitary CHRPE lesions have a typical depigmented ring or “halo” around the margin [13]. Congenital hamartoma of the RPE is characterized by a small para-foveal, deeply pigmented nodular lesion of a size of 1 mm in diameter and 1 to 1.25 mm in thickness that protrudes through the sensory retina, sometimes into the vitreous cavity. It has well-defined margins, minimal feeder vessels and subtle retinal traction [14]. A CHRRPE is a rare benign, unilateral and solitary lesion located at the posterior pole. It is characterized by an ill-defined gray retinal mass. It varies widely in size, ranging from 1 mm to more than 10 mm in diameter. It shows tortuous or straightened retinal blood vessels, probably due to secondary retinal traction from excessive glial tissue on its surface [15]. RPE adenomas are rare and can be benign or malignant. They are solitary, oval, unilateral, dark brown to black deep retinal tumors that invade over the sensory retina and acquire feeder vessels [13].

## Conclusion

This case report highlights an unusual presentation of a large pigment clump in a case of MacTel which has not been previously reported.

## Notes

### Competing interests

The authors declare that they have no competing interests.
